# Biomolecular alterations temporally anticipate microarchitectural modifications of collagen in oral tongue squamous cell carcinoma

**DOI:** 10.1016/j.isci.2024.110303

**Published:** 2024-06-17

**Authors:** Lucrezia Togni, Michele Furlani, Alessia Belloni, Nicole Riberti, Alessandra Giuliani, Valentina Notarstefano, Chiara Santoni, Elisabetta Giorgini, Corrado Rubini, Andrea Santarelli, Marco Mascitti

**Affiliations:** 1 Department of Clinical Specialistic and Dental Sciences, Marche Polytechnic University, via Tronto 10, 60126 Ancona, Italy; 2 Department of Life and Environmental Science, Marche Polytechnic University, via Brecce Bianche, Ancona, Italy; 3 Department of Neuroscience, Imaging and Clinical Sciences, University G. d’Annunzio of Chieti-Pescara, via dei Vestini 31, 66013 Chieti, Italy; 4 Department of Biomedical Sciences and Public Health, Marche Polytechnic University, via Tronto 10, Ancona, Italy; 5 Dentistry Clinic, National Institute of Health and Science of Aging, IRCCS INRCA, via Tronto 10, 60126 Ancona, Italy

**Keywords:** Biochemistry, Biophysics

## Abstract

High resolution analysis of collagen bundles could provide information on tumor onset and evolution. This study was focused on the microarchitecture and biomolecular organization of collagen bundles in oral tongue squamous cell carcinoma (OTSCC). Thirty-five OTSCC biopsy samples were analyzed by synchrotron-based phase-contrast microcomputed tomography and Fourier transform infrared imaging (FTIRI) spectroscopy. PhC-microCT evidenced the presence of reduced and disorganized collagen in the tumor area compared to the extratumoral (ExtraT) one. FTIRI also revealed a reduction of folded secondary structures in the tumor area, and highlighted differences in the peritumoral (PeriT) areas in relation with the OTSCC stage, whereby a significantly lower amount of collagen with less organized fibers was found in the PeriT stroma of advanced-OTSCC stages. Interestingly, no significant morphometrical mismatches were detected in the same region by PhC-microCT analysis. These results suggest that biomolecular alterations in the OTSCC stroma temporally anticipate structural modifications of collagen bundle microarchitecture.

## Highlights

• Intratumoral and peritumoral areas showed reduced and disorganized collagen bundles
• Biomolecular alterations anticipate structural collagen changes in the tumor stroma

## Graphical abstract

**Figure:**
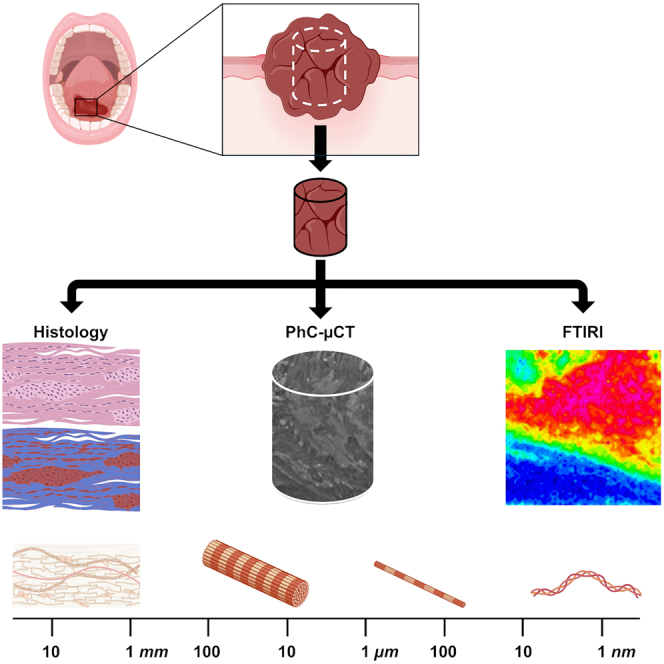

## Introduction

In recent years, there has been a growing interest toward the role of the extracellular matrix (ECM) in the biomechanics of physiologic/pathologic tissues. The ECM does not only surround cells, but its rigidity stresses them mechanically, producing signals that depend on both the amount of collagen and its cross-linking and hydration. It can also induce the so-called mechano-transduction process, namely cells convert a mechanical stimulus into an electrochemical activity. Collagen remodeling, including alterations in collagen fiber orientation, cleavage, and trimer composition, exerts pronounced effects on tumor growth and immunity, some of which are mediated by specific collagen receptors.^1^^,^^2^ In this light, the etiology of many types of pathologies, including tumors, could be elucidated by the characterization of the collagen bundle microstructure.

Oral tongue squamous cell carcinoma (OTSCC) accounts for over 40% of all oral cancer cases.^3^^,^^4^ It exhibits distinctive molecular and clinical behavior compared to oral cancer of other subsites leading to an “anatomical bias” both for research and clinical decision making.^5^ The stroma represents the main component of tumor microenvironment (TME) and plays a pivotal role in cancer progression and therapy resistance.^6^^,^^7^^,^^8^^,^^9^^,^^10^^,^^11^^,^^12^ It consists of non-malignant tumor cells and ECM; however, although stroma cells are not malignant themselves, the tumor-stroma interaction allows the acquisition of an abnormal phenotype.^13^

The temporal relationship between biomolecular alteration and structural modification of tumor stroma is still largely unknown: stroma alterations seem to occur before tumor is clearly detectable and have been shown to significantly predict poor patient outcomes.^10^^,^^14^ In particular, tumor stroma outgrowth is characterized by abnormal matrix secretion and collagen synthesis, attributable to hypoxia and chronic inflammation, leading to an edematous and unstable TME.^15^^,^^16^ To date, OTSCC pathological staging is based on morphological features of tumor parenchyma analyzed by conventional histological techniques, overlooking the importance of tumor stroma from a prognostic and therapeutic standpoint.

Recently, a variety of experimental methods quantify tumor characteristics from molecular to tissue scales, and describe how such data can be directly integrated with mechanism-based models to improve predictions of tumor growth and treatment response.^17^ Moreover, engineering tools—including advanced imaging techniques—were recently shown to improve our understanding of cancer ECM biology and therapeutic development.^18^ In this context, some authors of this paper recently investigated the morpho-chemical features of collagen bundles in uterine leiomyoma using an innovative synergistic approach based on two advanced imaging techniques, namely synchrotron-based phase-contrast microcomputed tomography (PhC-microCT) and Fourier transform infrared imaging (FTIRI) spectroscopy.^19^^,^^20^ Compared to the conventional X-ray imaging, synchrotron-based PhC-microCT allows the three-dimensional (3D) evaluation of the ECM microarchitecture with high spatial resolution and without using contrast agents.^20^ Moreover, FTIRI represents a versatile spectroscopic technique to obtain the morphochemical characterization of the same tissues, with additional information at macromolecular level.^21^^,^^22^

Based on this evidence, in this study we propose a multidisciplinary approach supported by synchrotron PhC-microCT and FTIRI spectroscopy to deeply investigate the IntraT, PeriT, and ExtraT regions of OTSCC at different pathological stages, focusing on the structural organization of ECM collagen bundles at multiscale. This investigation contributes to elucidate the temporal kinetics interlacing the stroma microarchitectural modifications and biomolecular alterations.

## Results

**Figure 1:**
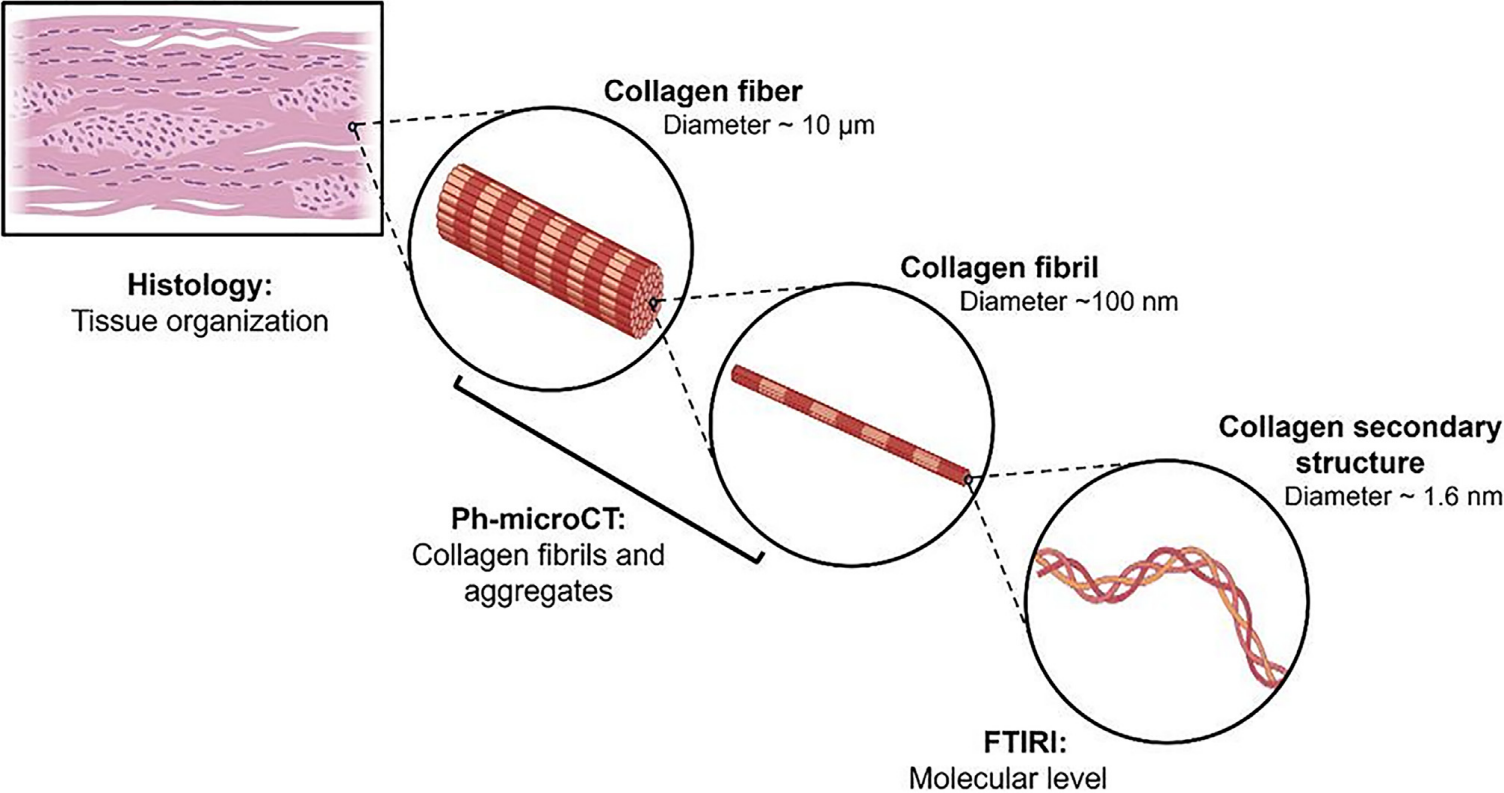
Structural and molecular organization of collagen Collagen is a fibrous protein composed of three left-handed alpha chains, wound together in a tight triple helix. Collagen molecules are aggregated to form fibrils and then fibers, which subsequently form the complex tissue organization.

The study was focused on biopsy samples with histological diagnosis of OTSCC at different malignancy stage (stages I–IV). Samples were investigated by PhC-microCT and FTIRI techniques. The flux of the study is described in Figure 1. The histopathological investigation of OTSCC biopsy samples was first carried out to identify specific areas containing the tumor mass (named IntraT area), the normal stroma (named ExtraT area) and the regions corresponding to the front of the tumor in each OTSCC stage (named PeriT S-I, Peri-T S-II, PeriT S-III, and Peri-T S-IV). From these areas, cylindrical portions were extracted to be analyzed by PhC-microCT and FTIRI analyses.

### Clinicopathological data of the patient’s cohort

A total of 35 surgical samples, corresponding to as many patients and stored in the Institutional Archive, were selected (Table 1), consisting of 23 males (65.7%) and 12 females (34.3%). The mean age at diagnosis was 64.4 ± 16.1 years and the mean tumoral diameter was equal to 3.1 ± 1.6 cm. According to the 8^th^ Edition of American Joint Committee of Cancer (AJCC) staging system, 7 patients (20.0%) were stage I, 8 (22.9%) were stage II, while 11 (31.4%) and 9 (25.7%) patients belonged to stages III and IV, respectively. According to 4^th^ Edition of Word Health Organization (WHO) Grading (G), most of the cases (51.4%) resulted poorly differentiated tumors (G3), 14 cases (40.0%) were moderately differentiated (G2), and 3 cases (8.7%) showed a well grade of differentiation (G1). The mean number of tumor buds (TB) was equal to 5.7 ± 6.7 and the mean value of tumor-stroma ratio (TSR) was 25.1% ± 22.4. According to the dichotomous scale, 6 cases (17.1%) recorded a low TSR, while 29 cases (82.9%) belonged to stroma-low group. The “immune-excluded” represented the most prevalent phenotype (18 cases), followed by the “immuno-inflamed” (12 cases) and the “immune-desert” (5 cases) groups. The predominant pattern of invasion-3 (PPOI) was the most prevalent pattern (42.9%), while the most of OTSCCs showed worst POI-3 (34.3%) (WPOI) and WPOI-4 (34.3%); no cases displayed WPOI-5.

**Table 1:** Main clinicopathological features of OTSCC surgical samples

Parameter	n. (%)	Mean ± SD (range)
Age		64.4 ± 16.1 (25–91)
Sex		
M	23 (65.7)	
F	12 (34.3)	
Diameter (cm)		3.1 ± 1.6 (0.3–7)
Margin		
Positive	18 (51.4)	
Negative	17 (48.6)	
Grading (4^th^ Ed. WHO)		
G1	3 (8.7)	
G2	14 (40.0)	
G3	18 (51.4)	
pT (8° Ed. AJCC)		
1	12 (34.3)	
2	17 (48.6)	
3	4 (11.4)	
4	2 (5.7)	
pN (8° Ed. AJCC)		
1	17 (48.6)	
2	10 (28.7)	
3	8 (22.9)	
Staging (8° Ed. AJCC)		
I	7 (20.0)	
II	8 (22.9)	
III	11 (31.4)	
IV	9 (25.7)	
WPOI		
1	8 (22.9)	
2	3 (8.7)	
3	12 (34.3)	
4	12 (34.3)	
PPOI		
1	10 (28.6)	
2	5 (14.3)	
3	15 (42.9)	
4	5 (14.3)	
Tumor budding		5.7 ± 6.7 (0–26)
TSR		
Stroma-rich	6 (17.1)	
Stroma-poor	29 (82.9)	
Immunophenotype		
Immune-inflamed	12 (34.3)	
Immune-excluded	18 (51.4)	
Immune-desert	5 (14.3)	

M, man; F, female; G, grading; PPOI, prevalent pattern of invasion; WPOI, worst pattern of invasion; TSR, tumor-to-stroma ratio; DS, standard deviation; WHO, World Health Organization; AJCC, American Joint Committee on Cancer; SD, standard deviation.

Locoregional recurrences occurred in 28.7% of cases after 24.5 ± 22.0 months from the initial surgical treatment, while 65.7% of patients died due to OTSCC with a disease specific survival equal to 27.6 ± 19.6 months.

### Histological analysis investigated the biopsy samples, highlighting areas containing both the tumor mass and the ExtraT region

**Figure 2:**
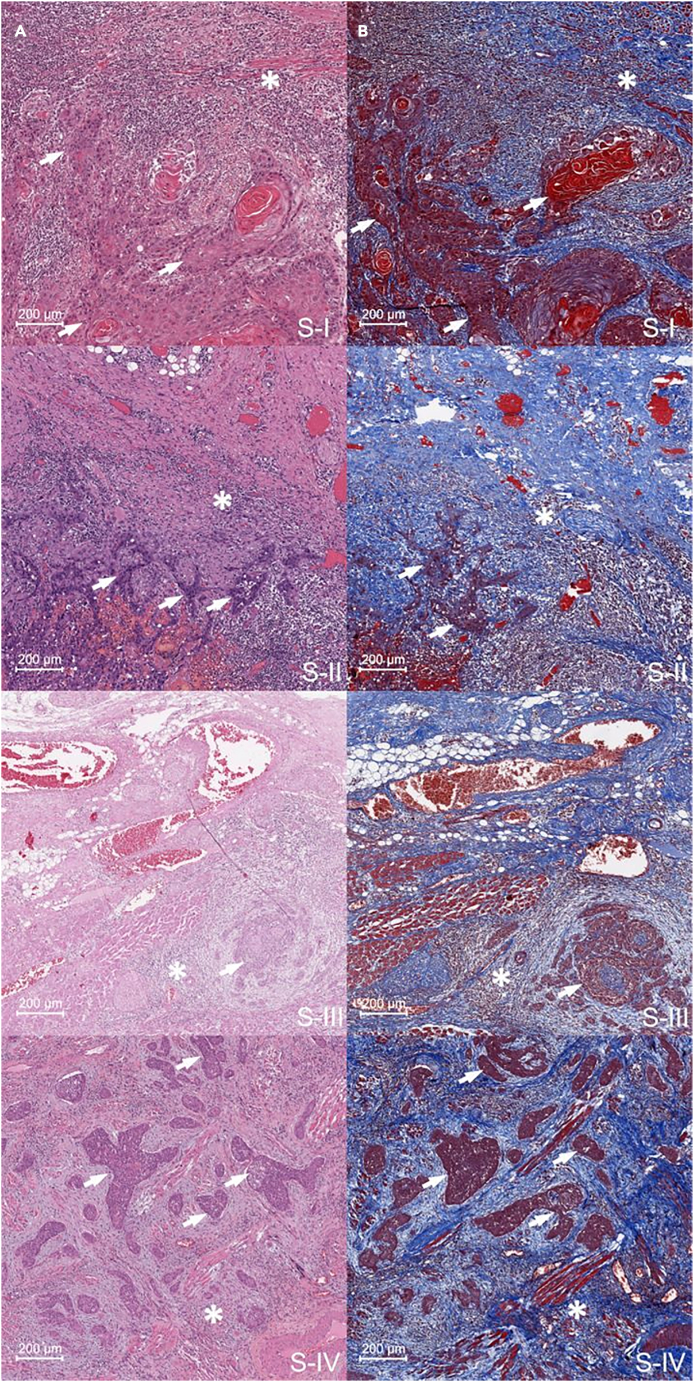
Examples of tumor and PeriT areas in histological sections of OTSCC (A) H&E and (B) Masson’s trichrome images (×20 magnification) of representative OTSCC sections showing the tumor areas (white arrows) and the PeriT stroma (white asterisks) at the tumor front in stages I (S-I), II (S-II), III (S-III), and IV (S-IV). A total of 70 histologic images (two for each biopsy) were investigated. Scale bars: 200 μm.

Biopsy samples were first submitted to histological analysis to identify and characterize the tumor regions and the areas surrounding the tumor itself; for each sample, Nr. 2 sections (4-μm thickness) were cut for hematoxylin & eosin (H&E) and Masson’s trichrome staining. As an example, in Figure 2, the histologic images of OTSCC sections representative of stages I–IV are reported displaying both the tumor mass and the PeriT stroma at the tumor front. The histological Masson trichrome images highlighted the presence of different areas corresponding to the tumor (red tones) and healthy stroma (blue tones) in the PeriT region. Modifications in tissue organization in the regions surrounding the tumor mass were evidenced, as displayed by a different collagen distribution.

### Synchrotron-based PhC-microCT evidenced significantly reduced and disorganized collagen in the tumor area but no significant morphometrical mismatches in the PeriT region between different OTSCC stages

**Figure 3:**
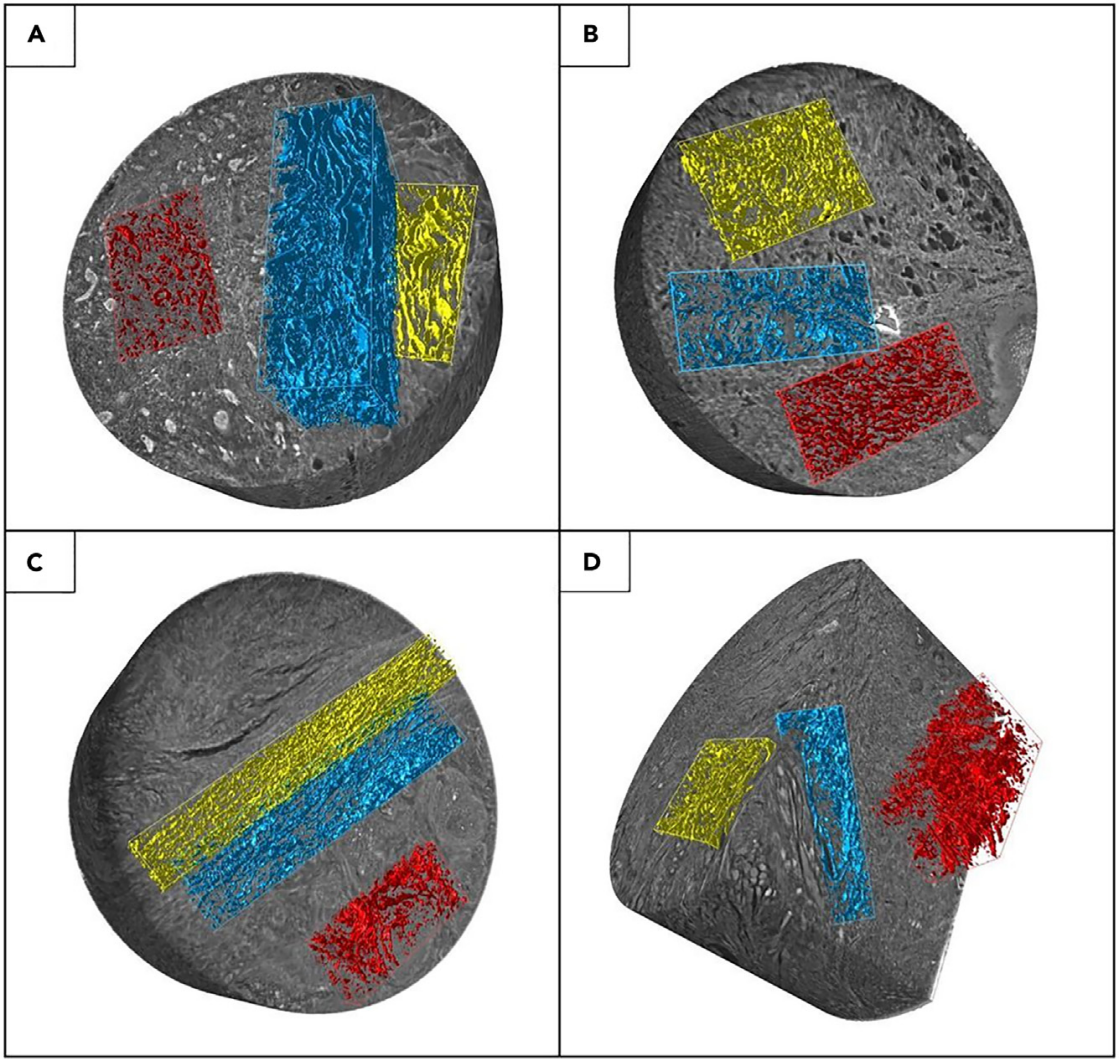
PhC-microCT 3D imaging of OTSCC 3D synchrotron-based PhC-microCT reconstruction of four representative OTSCC biopsies at (A) stage I, (B) stage II, (C) stage III, and (D) stage IV. Colored boxes highlighted the distribution of the collagen bundles in the IntraT (red), PeriT (blue), and ExtraT (yellow) areas. IntraT and PeriT regions seem characterized by loosely packed and randomly arranged collagen bundles regardless of the stage. Number of biopsies investigated: 35, one for each patient. Cylindrical samples: diameter ≅ 2 mm; height ≅ 3÷4 mm.

All the biopsies defined previously were sectioned to obtain the same number of cylindrical samples (diameter ≅ 2 mm; height ≅ 3÷4 mm) for high-resolution synchrotron tomography (pixel size 890 nm). The 3D reconstructions were analyzed; for each sample, three sub-volumes corresponding to the ExtraT area (normal stroma), the front of the tumor (PeriT stroma) and the tumor itself (IntraT stroma) were selected (Video S1) and divided according to the tumor stage. As displayed in Figure 3, 3D synchrotron imaging showed the presence of a dense and well-organized stroma (yellow false color) in the ExtraT area. Conversely, both the IntraT (red false color) and PeriT (blue false color) regions seemed characterized by loosely packed and randomly arranged collagen bundles, resulting in an edematous stroma.

Video S1. 3D synchrotron-based PhC-microCT reconstruction of a representative OTSCC sample, related to Figure 3 Colored boxes highlighted the intratumoral (red), peritumoral (blue), extratumoral (yellow) collagen bundles. Intratumoral and peritumoral regions are characterized by loosely packed and randomly arranged collagen bundles.

**Figure 4:**
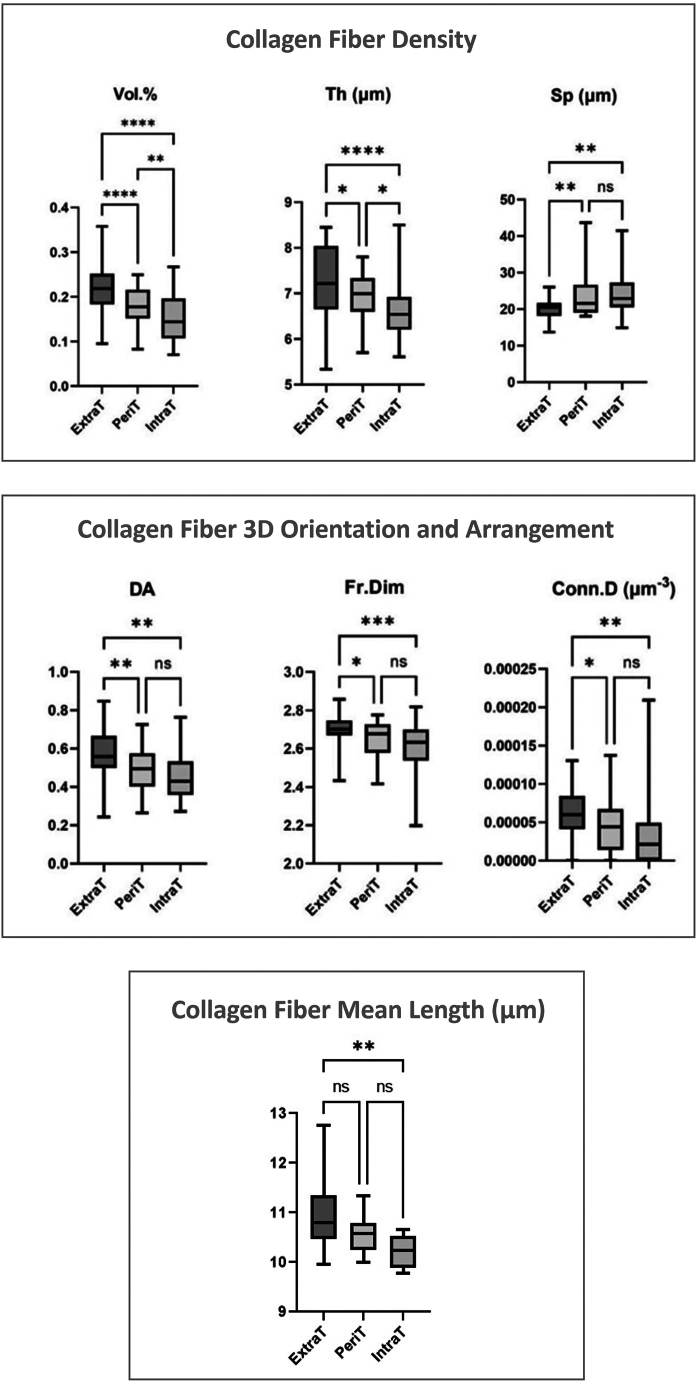
PhC-microCT data analysis Collagen morphometric parameters calculated in the extratumoral (ExtraT), peritumoral (PeriT), and intratumoral (IntraT) stroma of OTSCC samples. Box-and-whisker diagrams of the distribution of PhC-microCT morphometric parameters: connective tissue specific volume (Vol. %, [0÷1]), collagen bundle mean thickness (Th, μm), collagen bundle mean spacing (Sp, μm), connective tissue anisotropy degree index (DA, [0÷1]), connective tissue 3D fractal dimension (Fr.Dim, [2÷3]), connective tissue connectivity density (Conn.D, μm^−3^), collagen bundle mean length (mean fiber length, μm) Upper and lower ends of boxes represent 75^th^ and 25^th^ percentiles. Max and min values are reported. The median value is shown with a solid line. ∗*p* < 0.05; ∗∗*p* < 0.01; ∗∗∗*p* < 0.001; ∗∗∗∗*p* < 0.0001. Number of biopsies investigated: 35, one for each patient.

The statistical analysis, carried out on the complete set of samples, is reported in Figure 4, by box-and-whisker diagrams. It revealed the presence of a wide variability for all morphometric parameters. Repeated measures analysis of variance and Tukey’s multiple comparisons test evaluated the differences among extratumoral (ExtraT), peritumoral (PeriT) and intratumoral (IntraT) regions.

The volume percentage significantly increased from IntraT to PeriT and ExtraT stroma. Similarly, the ExtraT bundles were significantly thicker, both respect to PeriT and IntraT ones. Limitedly to these two parameters, statistically significant differences were also detected comparing PeriT and IntraT stroma. The spacing between bundles significantly decreased from ExtraT to PeriT and IntraT stroma.

Anisotropy degree, fractal dimension, and connectivity density were significantly higher in the ExtraT stroma compared to PeriT and IntraT ones. Interestingly, no significant differences (*p* > 0.05) were found between PeriT and IntraT stroma for spacing between bundles, anisotropy degree, fractal dimension, and connectivity density.

With reference to the mean fiber length, a significant deviation (*p* < 0.01) was detected between the ExtraT and IntraT regions of the same biopsy, with longer fibers on average in the ExtraT stroma. However, this result must be taken with caution due to the very high standard deviation influencing the mean fiber length within each individual biopsy. Indeed, the detected high standard deviation data confirm the broad heterogeneity of the tongue stroma, regardless of the presence or absence of OTSCC.

Moreover, one-way ANOVA and Tukey’s multiple comparison test did not reveal significant differences of the morphometric indices among the OTSCCs pathological stages in the PeriT stroma (data not shown).

### FTIRI evidenced a low amount of collagen—with reduced fiber organization in the tumor area as well as in the PeriT stroma of advanced-OTSCC stages

The same cylindrical samples (Nr. 35) analyzed by PhC-microCT were submitted to FTIRI analysis. In this regard, from each cylinder Nr. 2 sections (7-μm thickness) were cut and deposited onto CaF_2_ optical windows for the vibrational analysis. More in detail, by using the television camera, on each section, the areas containing the tumor mass, the normal stroma, as well as the front of the tumor were selected. On these areas, by using the focal plane array (FPA) detector, infrared (IR) images of 164 × 164 μm^2^ size were acquired; each image was the result of 4,096 pixel/spectra with a spatial resolution of 2.56 × 2.56 μm^2^.

**Figure 5:**
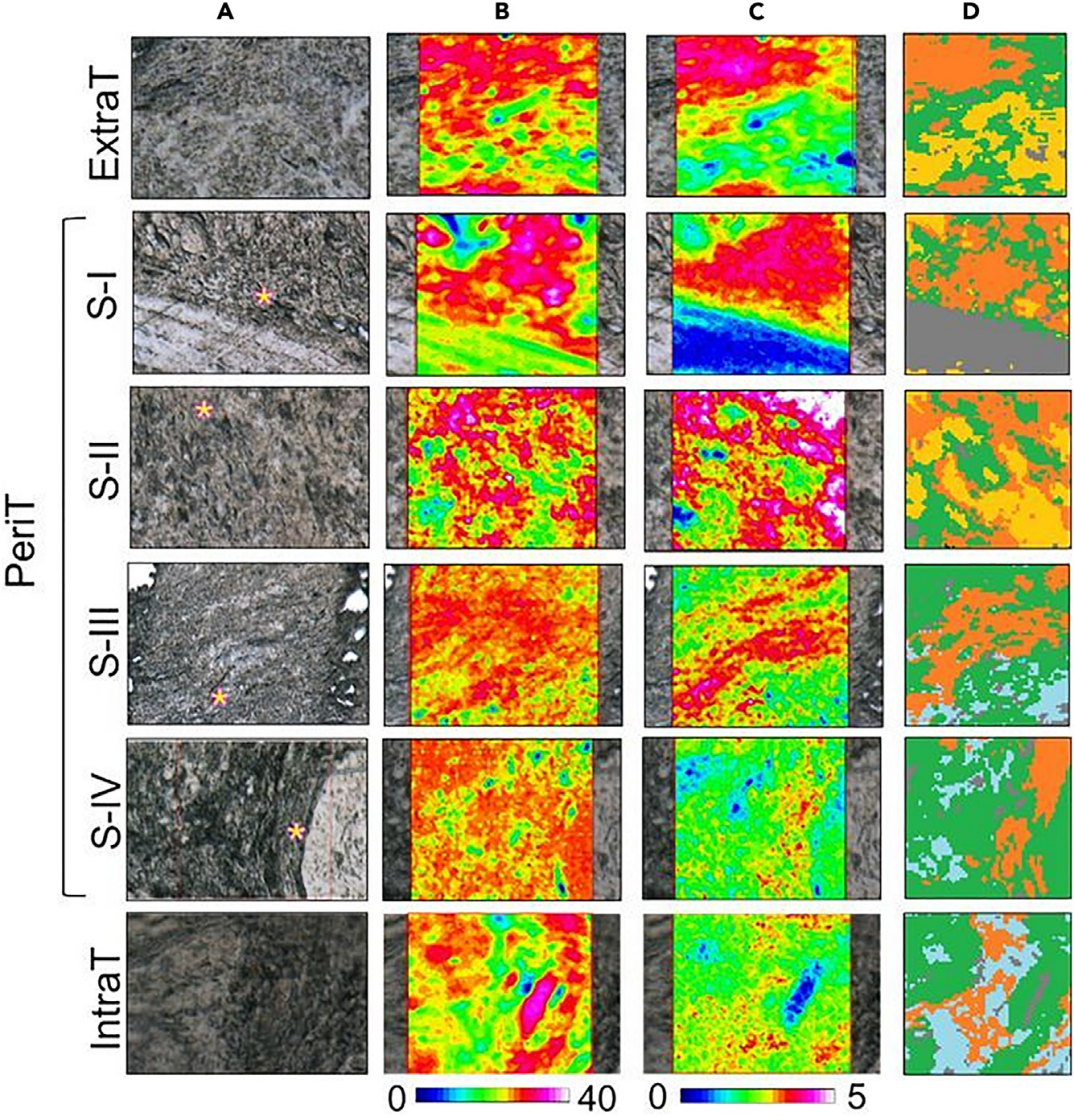
Hyperspectral imaging of OTSCC Hyperspectral imaging analysis of OTSCC sections representative of the extratumoral stroma (ExtraT), PeriT stroma near to OTSCC at stages I–IV (named respectively PeriT S-I, PeriT S-II, PeriT S-III, and PeriT S-IV), and IntraT stroma at stage IV (IntraT). (A) Microphotographs of the mapped areas (164 × 164 μm^2^ size, with 4,096 pixel/IR spectra; spatial resolution: 2.56 × 2.56 μm^2^; pink asterisks indicate the PeriT areas). (B and C) False color images showing the topographical distribution of (B) proteins (PROTEINS images) and (C) collagen (COLLAGEN images); false color images were built by using a specific color scale, with blue referring to areas with the lowest absorbance values, while white/pink to those with the highest ones; due to differences in the absorbance values, different scales were used for proteins and collagen. (D) Hierarchical cluster analysis (HCA) images (green color defining the areas with higher proteins amounts, while orange specifically indicating regions richer in collagen). Number of sections investigated: Nr. 70, two for each biopsy; on each section, an IR image was acquired respectively on the ExtraT, PeriT, and IntraT areas.

In Figure 5, the microphotographs of representative OTSCC sections, showing the ExtraT region (ExtraT), the PeriT areas near to OTSCC at stages I–IV (PeriT S-I, Peri T S-II, PeriT S-III, and PeriT S-IV) and the tumor mass at stage IV (IntraT) are displayed together with the false color images, and the corresponding hierarchical cluster analysis (HCA) images. False color images are useful to highlight the topographical distribution of specific biological compounds within a mapped area, in this case total proteins and collagen. To this purpose, they were generated by integrating IR images under the following spectral intervals: 1,720–1,470 cm^−1^ (which includes the amide I and II bands, representative of total proteins; PROTEINS images)^23^ and 1,299–1,184 cm^−1^ (which includes the amide III region, mainly representative of collagen; COLLAGEN images).^24^^,^^25^

Interestingly, the hyperspectral imaging analysis was able to differentiate each pathological stage. As regards total proteins (PROTEINS images, Figure 5B), an almost similar amount was found in all the analyzed samples, even if with a different distribution in relation with the tumor stage: in fact, total proteins displayed a more compact localization in the normal stroma (ExtraT) and in the PeriT region near to OTSCC at stage I (PeriT S-I), compared to the tumor mass (IntraT) and the PeriT areas close to OTSCC at stages II, III, and IV (PeriT S-II, PeriT S-III, and PeriT S-IV). Collagen appeared even more influenced by the pathological stage (COLLAGEN images, Figure 5C): (1) higher levels were found in the extratumoral region (ExtraT) and in the PeriT areas corresponding to early tumor stages (PeriT S-I and PeriT S-II); (2) interestingly, in these regions, a superimposable distribution of this matrix protein with total proteins was observed, allowing us to hypothesize that, in early tumor stages collagen is the main protein component like in normal stroma; (3) conversely, lower amounts of collagen were detected in the PeriT areas near to OTSCC at stages III and IV (PeriT S-III and PeriT S-IV), and in the intratumoral region (IntraT), with a different localization respect to proteins, suggesting a major contribution of tumor-related proliferating cells in advanced tumor stages.

**Figure 6:**
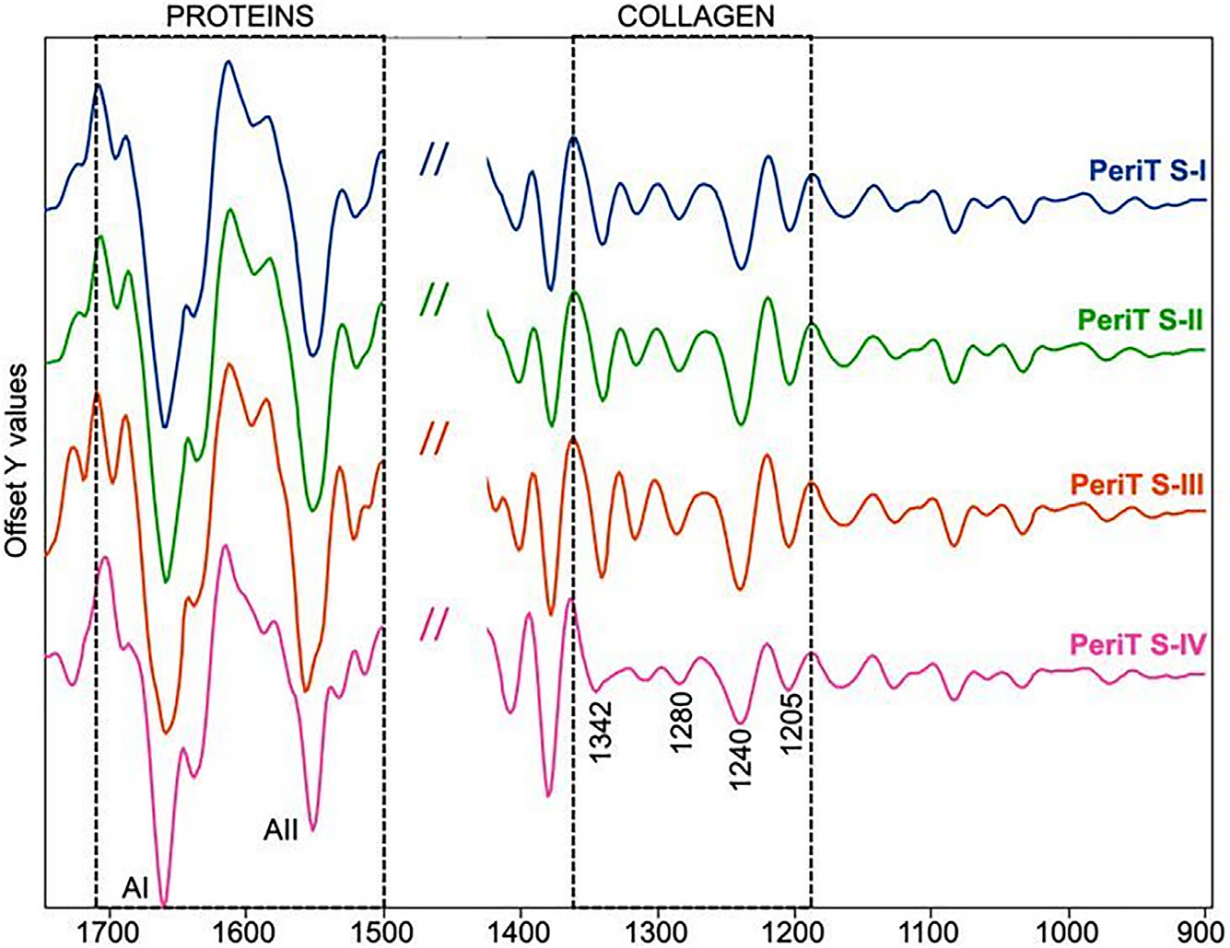
IR spectra of OTSCC PeriT stroma Average IR spectra representing the PeriT stroma in OTSCC samples from stages I–IV (named respectively PeriT S-I, PeriT S-II, PeriT S-III, and PeriT S-IV). IR spectra were extracted from collagen-rich clusters in HCA images and are reported in second derivative mode in the 1,720–900 cm^−1^ range to better highlight small differences among groups. The regions representative of proteins and collagen are indicated by dotted boxes and the position (wavenumbers, cm^−1^) of the main absorption peaks are reported.

IR images were also submitted to HCA to highlight, within the mapped areas, the presence of regions characterized by different spectral profiles and hence by a diverse macromolecular composition: in this study, HCA was useful to identify on each IR image collagen-rich clusters (orange colored in Figure 5D) from which to extract the average IR spectra (Figure 6).

Due to the presence of convoluted bands, typical of biological samples, IR spectra were submitted to peak fitting analysis, a procedure that made it possible to obtain the precise position (centroid, cm^−1^) of all peaks underlying a specific IR region together with the corresponding integrated areas. The analysis was focused on the amide I and II bands (centered respectively at ∼1,655 cm^−1^ and ∼1,550 cm^−1^), representative of total proteins, and on the amide III band (in the spectral interval 1,360–1,184 cm^−1^), mainly diagnostic for collagen.^24^^,^^26^^,^^27^^,^^28^ In particular, the following peaks were taken into account: ∼1,342 cm^−1^ (CH_2_ wagging of side chains in proline, the most recurrent amino acid in collagen); ∼1,319 cm^−1^ (α helix secondary structures); ∼1,280 cm^−1^ and ∼1,240 cm^−1^ (collagen triple helix); ∼1,262 cm^−1^ (random secondary structures), and ∼1,205 cm^−1^ (amino acids’ side chains).^19^^,^^26^^,^^29^

With this analysis, the following spectral parameters were calculated and statistically evaluated: collagen/proteins (COLL/PRT), calculated as ratio between the area of the spectral range 1,299–1,184 cm^−1^ (including the bands at 1,280 cm^−1^, 1,240 cm^−1^, and 1,205 cm^−1^, which form the tricuspid-shaped profile typical of collagen), and the area of the spectral range 1,720–1,470_cm^−1^ (including the amide I and II bands of proteins); proline/proteins (PRO/PRT), calculated as ratio between the area of the band at 1,342 cm^−1^ (corresponding to the wagging of proline) and the area of the spectral range 1,720–1,470 cm^−1^ (including the amide I and II bands of proteins); TRIPLE HELIX, calculated as ratio between the area of the band at 1,280 cm^−1^ (collagen triple helix) and the area of the range 1,360–1,184 cm^−1^ (representative of the total absorption in this interval); ALPHA HELIX, calculated as ratio between the area of the band at 1,319 cm^−1^ (alpha helix secondary structures) and the area of the range 1,360–1,184 cm^−1^ (representative of the total absorption in this interval); and RANDOM, calculated as ratio between the area of the band at 1,262 cm^−1^ (representative of random coil structures) and the area of the range 1,360–1,184 cm^−1^ (representative of the total absorption in this interval).^19^^,^^24^^,^^25^

**Figure 7:**
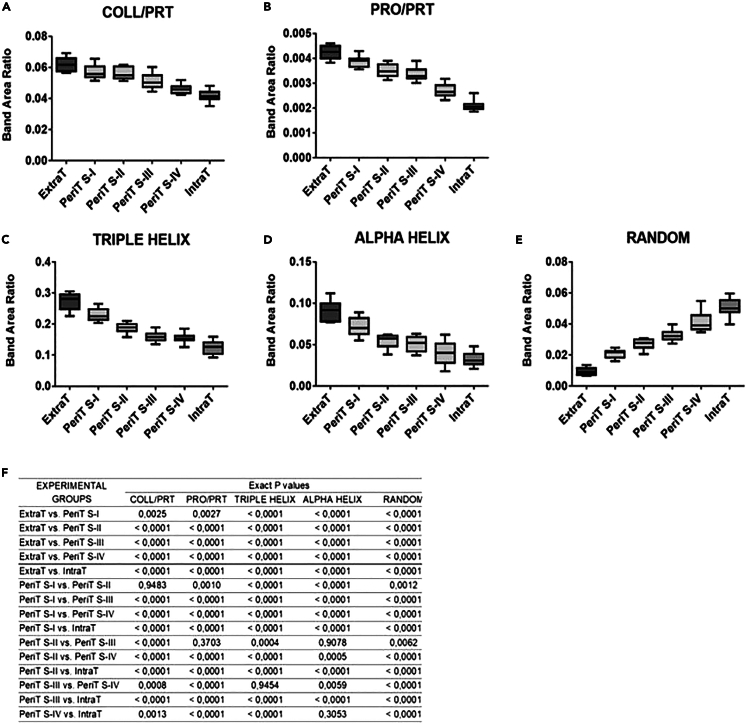
Statistical analysis of spectral parameters Statistical analysis of spectral parameters calculated in the extratumoral stroma (ExtraT), PeriT stroma near to OTSCC at stages I–IV (named respectively PeriT S-I, PeriT S-II, PeriT S-III, and PeriT S-IV), and intratumoral stroma at stage IV (IntraT). Box-and-whisker diagrams of: (A) COLL/PRT (representative of the relative amount of collagen respect to total proteins); (B) PRO/PRT (representative of the relative amount of proline respect to total proteins); (C) TRIPLE HELIX (representative of the total amount of the triple helix structures in collagen); (D) ALPHA HELIX (representative of the total amount of α-helix structures in collagen); (E) RANDOM (representative of random structures), and (F) *p* values. Upper and lower ends of boxes represent 75^th^ and 25^th^ percentiles. Max and min values are reported. The median value is shown with a solid line. Statistical significance was set at *p* < 0.05. Number of sections investigated: 70, two for each biopsy sample.

COLL/PRT and PRO/PRT ratios can both be considered representative of the relative amount of collagen, respect to total proteins (Figures 7A and 7B). As expected, the normal stroma (ExtraT) was characterized by the highest values of these two parameters, which decreased statistically significantly from PeriT S-I to PeriT S-IV, until reaching the lowest values in the tumor mass (IntraT). A similar statistically significant decreasing trend was displayed by TRIPLE HELIX and ALPHA HELIX ratios, representing respectively the relative amount of triple helix and α-helix structures in collagen (Figures 7C and 7D). Finally, the RANDOM ratio was analyzed to evaluate the structural organization of the protein component: the lowest values were found in the normal stroma (ExtraT), with an increasing trend going from PeriT S-I to PeriT S-IV and a major extent in the tumor mass (IntraT) (Figure 7E). These findings hypothesize a higher amount of collagen, characterized also by a major structural organization in the PeriT regions near the OTSCC in the early stages, similar to what is observed in the normal stroma; conversely, the tumor mass as well as the PeriT regions close to OTSCC at advanced stages are characterized by low levels of collagen, with a more disordered structure. *p* values are reported in Figure 7F; statistical significance among groups was set at *p* > 0.05.

### Correlation analysis among microarchitectural collagen bundles parameters, collagen macro-molecular indices, and clinical stages revealed that biomolecular alterations of collagen in the OTSCC stroma temporally anticipate structural modifications of collagen bundle microarchitecture

Spearman rank analysis focused on the PeriT stroma, correlating clinical staging with the most relevant microarchitectural parameters (Th, Sp, and Vol. %) of collagen bundles and with two macromolecular indices (i.e., COLL/PRT and PRO/PRT) (Table 2). The correlations were considered “very weak” (ρ = 0 ÷ 0.20), “weak” (ρ = 0.20 ÷ 0.40), “moderate” (ρ = 0.40 ÷ 0.60), “strong” (ρ = 0.60 ÷ 0.80), and “very strong” (ρ = 0.80 ÷ 1.0). “Strong” and “very strong” correlations with *p* < 0.05 were considered statistically significant.

**Table 2:** Spearman’s rank correlation analysis between the structural indices and the clinical-pathological data of the surgical samples in the PeriT stroma

	Stage	COLL/PRT	PRO/PRT	Th	Sp	Vol. %
Stage	ρ = 1	−0.60^a^	−0.77^a^	−0.25	−0.48	0.29
	*p* = 1	0.001^a^	0.000^a^	0.188	0.009	0.132
COLL/PRT		ρ = 1	0.28	0.63^a^	−0.03	0.49
		*p* = 1	0.147	0.000^a^	0.883	0.007
PRO/PRT			ρ = 1	0.07	0.44	0.43
			*p* = 1	0.702	0.016	0.020
Th				ρ = 1	0.18	0.48
				*p* = 1	0.344	0.009
Sp					ρ = 1	−0.69^a^
					*p* = 1	0.000^a^
Vol. %						ρ = 1
						*p* = 1

a Statistically significant associations (*p* < 0.05) with “strong” and “very strong” correlations. (COLL/PRT, collagen/proteins; PRO/PRT, proline/proteins; Th, mean collagen bundle thickness; Sp, mean collagen bundle spacing, Vol %: specific collagen volume).

Significant negative correlations between the pathological stage and both COLL/PRT (ρ = −0.60; *p* = 0.001) and PRO/PRT (ρ = −0.77; *p* < 0.0005) ratios were detected. Also, the microarchitectural Th parameter showed a significant direct correlation with the COLL/PRT ratio (ρ = 0.63; *p* < 0.0005) and a significant inverse correlation with the bundle spacing parameter (ρ = −0.69; *p* < 0.0005). Notably, no significant correlation was found between any of the collagen bundle microarchitectural parameters and the clinical staging (|ρ|<0.60, *p* < 0.05) in the PeriT stroma.

## Discussion

Solid tumors like OTSCC consist of the tumor parenchyma and stroma. The traditional view is that the tumor parenchyma regulates the surrounding stroma and promotes tumor progression. However, even though the pivotal role of the tumor stroma in assisting cancer growth and invasiveness is becoming increasingly clear, the precise mechanism underlying these processes is still not fully understood. The main histological features of OSCC staging are still referred to tumor parenchyma, and the temporal kinetics interlacing structural modifications of collagen bundles in the stroma and collagen biomolecular alterations in OTSCC staging are still mostly unexplored. In oral cancer, it is known that the tumor stroma is directly involved in the biological features of neoplastic cells.^30^^,^^31^^,^^32^^,^^33^^,^^34^ During oral tumorigenesis, an immature collagen and a higher level of proteolytic enzymes, neo-angiogenic, and profibrotic growth factors are released in the ECM, leading to the deposition of a reactive stroma.^35^^,^^36^ Moreover, the presence of a dysfunctional stroma has been significantly associated to unfavorable clinical outcomes in OTSCC patients,^37^^,^^38^^,^^39^^,^^40^^,^^41^ especially in early-OTSCCs^42^ This could be attributable to the reciprocal cross-talking between neoplastic cells and cancer-associated fibroblast (CAFs); indeed, high levels of stromal CAFs have been associated with a high risk of metastases and poor overall survival.^43^^,^^44^^,^^45^^,^^46^

However, there is still no solid evidence regarding the (temporal) cause-effect relationships linking the microarchitectural changes of the OTSCC stroma and the biochemical and macromolecular changes of the collagen protein. The main findings regarding tumor stroma in OTSCC is based on histological investigations, which are unable to quantitatively characterize the different portions of stroma due to the lack of both 3D view and high-resolution structural information of macromolecules in the IntraT, PeriT, and ExtraT areas. More reliable data can be obtained through investigations conducted on a large sample and through complementary techniques that provide significant information not only on the microstructure and 3D shape complexity of the collagen bundles but also on the macromolecular characteristics of collagen in the same analyzed tissues.

In this direction, in the present study we applied an innovative multidisciplinary and multiscale approach based on synchrotron PhC-microCT and FTIRI spectroscopy. We recently used the same cross-linked method to successfully study the massive collagen production in uterine leiomyoma^19^; hence, we proposed the same workflow, as it proved to be highly effective to investigate collagen self-organization in pathological conditions. The PhC-microCT morphometrical data revealed the presence of wide variability for all parameters, also in the ExtraT regions: this could be related to the structural heterogeneity of the stroma between the different collected areas among the patients, confirming that the tongue is an extremely heterogeneous anatomical site from a microarchitectural point of view. For this reason, statistical analysis was performed through a repeated measures analysis of variance, comparing each ExtraT area with the corresponding PeriT and IntraT ones (Figure 4). In terms of density, we observed and quantified the presence of a reduced amount of collagen bundles, thinner and more spaced in the proximity of the tumor parenchyma. This result could suggest the presence of an immature collagen (IntraT area), which is usually observed in infiltrative tumors, while a greater amount of thicker and closer collagen bundles should be attributable to a mature and physiological collagen (ExtraT area). In this direction, other authors confirmed the prevalence of thin collagen bundles in metastatic and advanced-stage tumors.^47^^,^^48^ Synchrotron imaging data also showed different arrangements of the collagen fibers between IntraT and ExtraT areas; indeed, it was demonstrated that the IntraT area has a greater isotropy, a smaller fractal dimension, and a very reduced connectivity compared to the ExtraT areas. This information, read together in the light of a biomechanical rationale, outlines evidence of compromised mechanical strength and tissue tension, and could explain the extreme dysfunctionality of the tumoral and PeriT stroma. Thus, in the PeriT and IntraT stroma, the collagen alterations could promote the clustering of the neoplastic cells in small nests, supporting the epithelial-to-mesenchymal transition (EMT). Synchrotron PhC-microCT results are corroborated by literature data; indeed, it has been demonstrated that collagen bundle arrangement undergoes a progressive disorganization from clinically healthy mucosa to advanced carcinomas.^49^^,^^50^

However, no significant morphometrical mismatches were found by PhC-microCT in the PeriT region between the different OTSCC stages. Conversely, FTIRI analysis showed significantly lower amounts of collagen in the PeriT stroma of advanced OTSCCs compared to early ones, characterized also by a significant reduction of folded secondary structures.

Moreover, the correlation analysis among microstructural indices, macro-molecular parameters, and clinical stage in the PeriT area of OTSCCs suggests the following observations: (1) there is a strong direct correlation between collagen bundle thickness and COLL/PRT ratio; (2) there are strong inverse correlations between stage and both COLL/PRT and PRO/PRT ratios; (3) no relevant (“strong” or “very strong”) correlation is present between the clinical stage and the microarchitectural collagen bundle parameters (bundle thickness, inter-spacing, and volume percentage).

These data support the synchrotron results that did not detect any significant difference between the pathological stages in collagen bundle microarchitectural organization. Conversely, the FTIRI analysis showed, at a finer (macromolecular) scale level, the presence of collagen that progressively decreases with the progression of the clinical stages.

To the best of our knowledge, this evidence suggests that during tumor progression, the macromolecular deviations anticipate, at a temporal level, the coarser 3D-organization modifications of the collagen bundles present in the stroma. Indeed, collagen type I (Col-I) degradation seems to be the crucial step during the stepwise oral cancer tumorigenesis, as the main target of MMPs in desmoplastic tumors. In particular, literature data showed a significantly higher expression of MMP-1, -2, -3, and -9 in the PeriT stroma of advanced OSCCs.^51^^,^^52^^,^^53^^,^^54^^,^^55^^,^^56^ Moreover, MMP-3 was found to be strongly expressed in the peripheral borders of invasive tumor islands of OSCC samples, where collagen fibers appear to be disrupted and degraded.^57^ In fact, in the PeriT stroma, the stromal progenitor cells are constantly recruited and CAFs produce a greater level of proteases, promoting the ECM degradation. In addition, MMPs can release bioactive fragments associated with other critical pathways, promoting angiogenesis, tumor proliferation, and migration. Therefore, the ECM proteolytic remodeling and the activation of a migratory cellular phenotype could represent a possible mechanism by which the MMPs were associated with poor survival outcomes. A recent animal model study demonstrated that the growth and metastasis of pancreatic cancer cells depend on Col-I cleavage that activates discoidin domain receptor 1 (DDR1) signaling. It has been shown that MMP-cleaved COL-I (cCOL-I) fibers promote pancreatic ductal adenocarcinoma (PDAC) bioenergetics and the tumor growth and metastasis, so that PDAC patients with over-expression of cCOL-I and lower expression of DDR1 showed poor survival outcomes. Therefore, the COL-I cleavage status could represent an important prognostic indicator for cancer patients.^58^^,^^59^

In conclusion, these data could provide a significant contribution to understanding the TME rearrangement during oral tumorigenesis, in order to better comprehend the relationship between neoplastic epithelium and tumor stroma. Moreover, the morphometrical analysis of staging parameters and adverse risk factors could be facilitated.

### Limitations of the study

Having learned in the present study that biomolecular alterations temporally anticipate microarchitectural changes of the stroma in OTSCCs, studying the intermediate hierarchical dimensions of collagen, from secondary to quaternary, in healthy and OTSCC contexts would be necessary to better understand the entire pathological process and its effects on the mechanotransduction of biochemical signals. This limitation of the present study will be overcome in follow-up investigations, which will be performed via second harmonic generation microscopy.

## Ethics statement

The study received ethical approval from the institutional review board of Marche Polytechnic University (CERM 2019-308) and was conducted in accordance with the Helsinki Declaration.^60^

## STAR★Methods

### Key resources table

**Table:** 

REAGENT or RESOURCE	SOURCE	IDENTIFIER
**Biological samples**		

OTSCC tissue samples	Pathology Institute, Marche Polytechnic University	CERM 2019-308

**Chemicals, peptides, and recombinant proteins**		

Mayer’s Hematoxylin	Bio-Optica	05-M06002
Eosin	CARLO ERBA Reagents	446664
Masson's Trichrome Stain Kit, Artisan™	Agilent Dako	AR17392

**Software and algorithms**		

SYRMEP Tomo Project software	Elettra Sincrotrone Trieste SCpA	https://github.com/ElettraSciComp/
Dragonfly software	Comet Technologies Canada Inc.	https://www.theobjects.com/dragonfly/index.html
ImageJ software v.2.9.0 – FIJI package	Schindelin et al.^61^	https://imagej.net/ij/
OPUS software v.7.5	Bruker Optics	https://www.bruker.com/
CytoSpec software v.2.00.01	Peter Lasch	https://www.cytospec.com/
GRAMS/AI v.9.1	Galactic Industries, Inc.	https://www.gramssuite.com/
GraphPad Prism v.8.0	GraphPad Software	https://www.graphpad.com/
SPSS statistical software v.25.0	IBM Corporation	https://www.ibm.com/spss

### Resource availability

#### Lead contact

Further information and request for resources and reagents should be directed to and will be fulfilled by the lead contact, Alessandra Giuliani (a.giuliani@staff.univpm.it).

#### Materials availability

The study did not generate new unique reagents.

#### Data and code availability

• All data reported in this paper will be shared by the lead contact upon request.
• This paper does not report original code.
• Any additional information required to reanalyze the data reported in this paper is available from the lead contact upon request.

### Experimental model and study participant details

#### Data collection and preparation

The study included patients with histological diagnosis of OTSCC, surgically treated with curative intent at the Department of Maxillofacial Surgery, “Ospedali Riuniti” General Hospital, Ancona, Italy, between 1997 and 2018. A total of 35 patients (23 males and 12 females) of Caucasian ethnicity, all Italian from the Marche region, were included; further information related to the participants can be found in the Results section. Informed consent was obtained from all patients and the study received ethical approval from the institutional review board of Marche Polytechnic University (CERM 2019-308). It was conducted in accordance with the Helsinki Declaration^60^ and BRISQ checklist (Supplemental Materials).^62^ The clinicopathological data were collected from the archive of the Pathology Institute, Marche Polytechnic University, Italy, and from clinical records, by a single examiner to ensure their uniformity. Inclusion criteria were primary OTSCC (International Classification of Disease-10: C02.0, C02.1, C02.2 e C02.3), age ≥ 18 years, no preoperative chemo- or radiation therapy, no human papilloma virus infection (assessed by HPV 16-specific fluorescence *in situ* hybridization and p16Ink4a-specific immunohistochemistry), and follow-up data of at least 3 years for living patients. Exclusion criteria were oral cancers involving other anatomical subsites, OTSCC with no precise identification of the origin site, and relapsed or secondary OTSCCs. The surgical samples were further stratified to allocate the similar number of patients in each pathological stage group (I, II, III, and IV). Recall for the updating of follow-up data was made by phone call by a single operator, blinded to clinicopathological data and group allocation. All patients had postoperative follow-up every 2 months for the first year, every 3 months during the second year, and every 6 months thereafter. If a patient had symptoms or signs of suspected recurrence, an immediate postoperative visit was scheduled.

### Method details

#### Histological analysis

To confirm the original diagnosis, for each case, 4-μm thick serial sections were cut from formalin fixed, paraffin-embedded (FFPE) blocks, which included the most invasive part of the primary tumor (the same routinely used to assess the depth of invasion status).

To confirm the original diagnosis and to assess the quality of the biospecimens, each sample was histologically revaluated and reclassified by 2 expert pathologists, blinded to the clinicopathological data, according to the 8th Edition of American Joint Committee on Cancer (AJCC) Cancer Staging Manual^63^ and 4^th^ Edition of Word Health Organization (WHO) Classification of Head and Neck tumors.^64^

The histopathological evaluation of the morphological parameters was performed by the oral pathologists, on the Haematoxylin (Bio-Optica, id.05-M06002, Milan, Italy) and eosin (CARLO ERBA Reagents, id.446664, Milan, Italy) (H&E) stained sections, using an Olympus BM50 optical microscope (Olympus, Tokyo, Japan). Any disagreement between the pathologists was resolved by unanimous consensus. For each case, the following morphological parameters were evaluated: (a) Pattern of invasion, according to Brandwein-Gensler et al. [64]: POI was evaluated at low magnification (×4) along the infiltrative tumor front. POI-1 was assigned to tumors with pushing, well-delineated infiltrating borders; POI-2 to tumors with infiltrating, solid cords, bands and/or strands; POI-3 to tumors with small groups or cords of infiltrating cells (n>15); POI-4 to tumors with marked and widespread cellular dissociation in small groups of cells (n<15) and/or in single cells; POI-5 to tumors with tumour satellites of any size ≥1 mm away from main tumour or next closest satellite with intervening normal tissue. Furthermore, for each sample, the WPOI and the PPOI were scored. For the WPOI no minimal cut-off value was established; (b) Tumor budding: the tumoral infiltrative front was initially scanned at low magnification (×4) to select the area with the greatest number of budding foci (“hotspot area”). Then, the number of TB was counted in the hotspot area at higher magnification (×20); (c) Tumor stroma ratio, according to Mascitti et al.,[6]: the tumoral infiltrative front was initially scanned at low magnification (×4) to identify the most invasive area with the highest percentage of stroma. Later, the same area was evaluated at high magnification (×20) to select a single high-power field where tumour cell nests were present at all borders of the selected image field. The percentage of stroma tissue was manually estimated per 10-fold, per image field; subsequently, patients were dichotomised in two groups, considering the optimal threshold of neoplastic tissue percentage, determined by Mesker et al.[65]: a stroma-rich group (TSR<50%); and a stroma-poor group (TSR≥50%); and (d) Immunophenotype: The density and localization of tumor-infiltrating lymphocytes (TILs) were determined based on the recommendation of the International TILs Working Group. For each sample, the H&E-stained section was fully assessed at low magnification (x4). Subsequently, the presence of TILs was evaluated in the stromal compartment of five random high-power fields (×20), considering all the percentage of the area occupied by mononuclear cells within the borders of the invasive tumor. The area percentage was estimated per 10-fold per image-field, and the mean value of scored percentages was considered for each sample[66]. Based on the results, patients were divided into the following groups: “immune-inflamed” phenotype (the mean percentage of lymphocytes inside the tumor mass in proximity to the tumor cells was ≥10%, regardless the percentage of stromal TILs around the tumor border); “immune-excluded” phenotype (the mean percentage of stromal lymphocytes around the tumor border was ≥10% with a negligible amount of intratumoral lymphocytes [<10%]); and “immune-desert” phenotype (the mean percentage of lymphocytes detected both in the tumor mass and in the stromal area was negligible [<10%])[4].

Furthermore, each sample was also submitted to histochemical analysis using Masson’s Trichrome staining (Masson's Trichrome Stain Kit, Artisan™, AR17392, Agilent Dako) to characterize the main components of the connective tissue: the staining highlights the presence of muscle (red-staining), collagen (blue-staining), fibrin (pink staining), erythrocytes (red-staining) and nuclei (blue/black-staining).

#### Synchrotron radiation-based PhC-microCT

The histological analysis also allowed to highlight areas containing both the tumor mass and the extratumoral region. Hence, for each biopsy, a cylindrical sample (diameter ≅ 2 mm; height ≅ 3÷4 mm) was cut from the most invasive area with the highest stroma percentage using a 13-gauge bone-marrow transplant needle (Hospital Service SpA, Aprilia, Italy).

All the nr.35 cylindrical samples were investigated by PhC-microCT scanning, which was performed at SYRMEP beamline, ELETTRA Synchrotron Facility, Trieste, Italy, using the white beam and the following settings: silicon filter (thickness = 0.5 mm); peak energy = 17 keV; number of projections/180°: 1800; angular step = 0.1°; exposure time/single projection = 0.2 s; sample-detector distance = 100 mm; pixel size = 890 nm. Data acquisition was scheduled in a single session to avoid image biases related to different working cycles.

The tomographic reconstruction was performed at the Physics Laboratory, Marche Polytechnic University, Italy, using the SYRMEP Tomo Project software,^65^ through the Paganin’s method.^66^ The δ/β ratio was set to 100. For each sample a volume of 6 mm^3^ (2048×2048×2048 pixel^3^) was acquired and reconstructed. Subvolumes of interest (VOI - 0.1 mm^3^) were extrapolated both from the peritumoral, intratumoral and extratumoral stroma, using 3D-visualization and the orthographic projections in the Dragonfly software (v.2022.1, ORS, Montréal, Quebec), and pre-processing with the Frangi3D filter. The 3D microstructural analysis was performed using the BoneJ tool of FIJI (ImageJ v.2.9.0)^61^ to characterize the following morphological indices: the collagen specific volume (Vol.%), measuring the collagen density, i.e. the ratio between the collagen volume and the total volume of the investigated VOI; the mean collagen bundle Thickness (Th; μm) and the mean collagen bundle Spacing (Sp; μm), measuring the mean distance between two collagen bundles. The 3D orientation and arrangement indices (namely, the Anisotropy Degree (DA), the Connectivity Density (Conn.D; μm^-3^), and the Fractal Dimension (Fr.Dim)) were also calculated. DA was used to quantify the directionality of the collagen bundles; it evaluates whether the bundles have a certain orientation, or if they’re randomly aligned. The method to measure anisotropy is fairly complex and consists of multiple steps: find mean intercept length (MIL) vectors from n directions; plot MIL vectors into a point cloud; solve the equation of an ellipsoid that best fits the point cloud; calculate the degree of anisotropy from the radii of the ellipsoid. DA = 0.0 means the image is completely isotropic, thus the sample has no directionality whatsoever; DA = 1.0 means there is an extreme prevailing orientation in the structure of the image. Conn.D parameter is designed to estimate the number of connected structures i.e. bundles in a network. This connectivity measure is related to a topological number χ known as Euler number. Mathematically, connectivity is defined =1−(χ+Δχ), where χ describes the shape or structure of a topological space and the term Δχ corrects for the change in the topology of an object, when it is cut to pieces; the input image must be 3D and binary; the resulting parameter is the Connectivity density (Conn.D): number of elements per unit volume, showing higher values for better-connected collagen bundles and lower values for poorly connected ones. The fractal dimension (FrD) parameter estimates the fractal dimension of a stack of images by applying the box-counting algorithm. In this algorithm grids of diminishing size are scanned over the image, and the number of boxes containing at least one foreground voxel is counted. As the box size decreases and the grid becomes finer, the proportion of foreground boxes increases in a fractal structure. In 3D the FrD value ranges from 2 (planar distribution) to 3 (fully 3D distribution).The box-counting algorithm, with the following starting setup was used^67^: box initial size [px]: 48; smallest box size [px]: 6; box scale factor: 1.2; grid translation: 0.

The mean Fiber Length was studied using the BoneJ Analyze Skeleton tool after removing the noise and binarizing.

#### Fourier Transform InfraRed Imaging

FTIRI measurements were carried out at the Advanced Research Instrument (ARI) Laboratory, Department of Life and Environmental Sciences, Marche Polytechnic University, by a Bruker INVENIO-R interferometer coupled with a Hyperion 3000 Vis-IR microscope and a Focal Plane Array detector (Bruker Optics, Ettlingen, Germany). The FPA detector is an array detector, usually applied for high resolution spectral imaging analysis of non-homogeneous samples, such as tumoral tissues.

Overall, Nr. 2 sections (7-μm thickness) were cut from each cylindrical sample and deposited onto CaF_2_ optical windows. On each section, IR images were acquired in transmission mode in the intratumoral, peritumoral, and extratumoral regions (15× condenser/objective; 4000–900 cm^-1^ spectral range; spectral resolution 4 cm^-1^; 256 scans). Each image was sized 164×164 μm^2^, and contained 4096 pixels/spectra, with a spatial resolution of 2.56×2.56 μm^2^. Before each acquisition, the background spectrum was collected with the same setup on a clean portion of the CaF_2_ optical window. All IR images were pre-processed to avoid the contributions of atmospheric carbon dioxide and water vapour, and vector normalized to correct variations in section thickness (respectively Atmospheric Compensation, and Vector Normalization routines; OPUS 7.5 software package, Bruker Optics, Ettlingen, Germany).

On pre-processed IR images, the topographical distribution of proteins and collagen was highlighted by integration under the following spectral regions: 1720–1470 cm^-1^ (PROTEINS images) and 1299–1184 cm^-1^ (COLLAGEN images). Different color scales were used: blue color referring to areas with the lowest absorbance values, while light pink/white to regions with the highest ones. Pre-processed IR images were also subjected to Hierarchical Cluster Analysis (HCA), using Euclidean distance and Ward's method (CytoSpec software v. 2.00.01). The average IR spectra were extracted from the collagen-rich areas identified by HCA and submitted to peak fitting analysis in the 1720–1470 cm^-1^ and 1360–1184 cm^-1^ spectral ranges (Euclidean distance; Gaussian function) (GRAMS/AI 9.1, Galactic Industries, Inc., Salem, New Hampshire). For each underlying peak, the position detected by Second Derivative minima analysis and expressed as wavenumbers, and the integrated area was obtained.

Spectral data were used to calculate the following band area ratios: COLL/PRT (ratio between the area of the spectral range 1299–1184 cm^-1^ and the area of the spectral range 1720–1470 cm^-1^); PRO/PRT (ratio between the area of the band at 1342 cm^-1^ and the area of the spectral range 1720–1470 cm^-1^); RANDOM (ratio between the area of the band at 1262 cm^-1^ and the area of the range 1360-1184 cm^-1^); TRIPLE HELIX (ratio between the area of band at 1280 cm^-1^ and the area of the range 1360-1184 cm^-1^), and ALPHA HELIX (ratio between the area of the band at 1320 cm^-1^ and the area of the range 1360-1184 cm^-1^).

#### BRISQ report for histology, PhC-microCT, and FTIRI

Regarding sample acquisition, collection, stabilization, and preservation for histological analysis, the following protocol was conducted in accordance with standard operating procedures adopted by Pathology Unit, Department of Biomedical Sciences and Public Health, Marche Polytechnic University, Ancona, Italy (n. protocol: PO01.IO03.AP) and with Italian Ministry of Health Guideline regarding management of materials for diagnostic purposes in pathology (REF: https://www.salute.gov.it/portale/documentazione/p6_2_2_1.jsp?lingua=italiano&id=2369). More detailed information about standard operating procedures is available to others upon request (Prof. C. Rubini, mail: corrado.rubini@ospedaliriuniti.marche.it).

Surgical samples of Oral Tongue Squamous Cell Carcinoma (OTSCC) came from patients treated with a curative intent at “Ospedali Riuniti” General Hospital (Ancona, Italy). The *in vivo* ischemic time (i.e., the time from the interruption of the blood supply to the tumor by the surgeon to the excision of the tissue specimen) was not known, although it never exceeded 3 hours. After the complete excision of tumor tissue, the surgical samples were sent within 30 minutes to the laboratories of the Pathology Unit in a standardized container to conduct an intraoperative pathology examination of the resection margins. The masses of the tumor tissue were then subjected to fixation in 10% neutral buffered formalin (NBF), with an *ex vivo* ischemic time (i.e., the time from excision to the initiation of tissue fixation) within 30 minutes. During this phase, surgical samples were exposed to a room temperature range of 19-22°C.

Samples were fixed at room temperature range of 20-25°C for 24-48 hours in 10% NBF formalin, in order to ensure complete fixation of the tissue samples. After that, samples were embedded in paraffin wax through Leica ASP6025 S tissue processor (Leica Biosystems Nussloch GmbH, Nussloch, Germany), Bio-optica BEC 150 paraffin embedding station and Bio-optica BTP 170 cooling plate (Bio-Optica SpA, Milan, Italy) according to the following protocol:

**Table:** 

Step	Reagent	Time (min:sec)
1	70% ethanol	30:00
2	80% ethanol	30:00
3	95% ethanol	30:00
4	100% ethanol	30:00
5	100% ethanol	60:00
6	100% ethanol	90:00
7	Xylene	45:00
8	Xylene	45:00
9	Xylene	90:00
10	Paraffin	60:00
11	Paraffin	60:00
12	Paraffin	90:00

After that, formalin-fixed, paraffin-embedded (FFPE) blocks of maximum size 32 × 25 × 6 mm were obtained, each containing a tissue sample of maximum size 25 × 20 × 4 mm.

For the first six months after preparation, paraffin tissue sections are stored in dedicated boxes in a temporary archive located within the Pathology Unit, characterized by a temperature (<27°C) and humidity (30-70%) controlled environment. After that, paraffin tissue sections are transferred for definitive storage (>10 years) by an operator into dedicated containers in an archive located within the “Ospedali Riuniti” General Hospital, characterized by a temperature (19-24°C) and humidity (40-60%) controlled environment. During transport, lasting <15 min, the samples are exposed to the temperature and humidity conditions of the environment, in accordance with the standard operating procedures.

To confirm the original diagnosis and to assess the quality of the biospecimens relevant to the subsequent analysis, such as adequate amount of tissue in the FFPE block (minimum size 15 × 10 × 2 mm) and absence of extensive areas of necrotic or hemorrhagic tissue, each sample was histologically revaluated before experimental analysis by 2 expert pathologists. For each case, 4-μm serial sections from FFPE blocks were carried out by Leica RM2125 RTS rotary microtome (Leica Biosystems) until obtaining slices in which the whole area of the tissue is present. By using a brush, the slices were placed in a tray with cold water and then on microscope slides in a thermostatic bath at the temperature of 46°C. Finally, the slides were placed on a dedicated basket in a thermostat at 50-57°C until completely dry. Serial sections were obtained from FFPE blocks including the most invasive part of the primary tumor (i.e., the same routinely used to assess the depth of invasion status). Before slides were coverslipped through Leica CV5030 coverslipper (Leica Biosystems), hematoxylin-eosin-stained sections were obtained through Leica Autostainer XL (ST5010) automated slide stainer (Leica Biosystems) according to the following protocol:

**Table:** 

Step	Reagent	Time (min:sec)
1	Oven (65°C)	10:00
2	Xylene	2:00
3	Xylene	2:00
4	100% alcohol	2:00
5	100% alcohol	2:00
6	70% alcohol	1:00
7	Wash medium	2:00
8	Hematoxylin	5:00
9	Wash medium	2:00
10	HCl alcohol	0:02
11	Wash medium	3:00
12	Scott’s medium	3:00
13	Wash medium	3:00
14	Eosin	2:00
15	95% alcohol	0:30
16	100% alcohol	2:00
17	100% alcohol	2:00
18	100% alcohol	2:00
19	Xylene	1:00

Regarding sample acquisition, collection, stabilization, and preservation for PhC-microCT analysis, cylindrical sections from FFPE blocks, stored in a dedicated box and with temperature (<27°C) and humidity (30-70%) controlled environment away from light sources, were brought to the Italian Phase Contrast Imaging Flagship Node (SYRMEP beamline of the ELETTRA Synchrotron Facility, Trieste, Italy) for data acquisition. The experiment received the availability for 9 shifts, able to analyze the 36 surgical specimens in 72 hours, including the technical times and the optimization of settings. For each PhC-microCT acquisition, one sample was fixed to the metallic flat plate (sample holder) with a fixing vax at the lower base of the specimen. All the scans were performed using white X-ray beam (peak energy = 17 keV) filtered with a silicon plate (thickness = 0.5 mm); the exposure time per projection was set to 0.2 s, with a collection of 1800 projections over a total range of 180°; the sample-detector distance was set to 100 mm, resulting in a pixel size of 890 nm. Data acquisition was conducted from 10 to 12 September 2021. After each sample acquisition, it was removed by the users and stored again in the dedicated box with controlled environment.

Regarding sample acquisition, collection, stabilization, and preservation for FTIRI analysis, cylindrical sections from FFPE blocks were brought from the Italian Phase Contrast Imaging Flagship Node (Trieste, Italy) to the Department of Life and Environmental Sciences, Polytechnic University of Marche (Ancona, Italy), stored in a dedicated box and with temperature (<27°C) and humidity (30-70%) controlled environment away from light sources. All phases of FTIRI analysis were performed in a room with controlled temperature (19°C). The specimens were embedded again in paraffin to make easier the cut of the sections that would be submitted to FTIRI analysis. The procedure involved the use of liquid paraffin to embed the cylindrical sections in a larger device without dissolve the pre-existing embedding, and hence without undergone the OTSCC sample to further manipulations. After the solidification of the wax, the re-embedded OTSCC blocks were stored at room temperature in dedicated glass containers before proceeding with their cut.

For FTIRI analysis, for each FFPE sample, 7-μm serial sections from paraffin embedded blocks were cut at ∼150 μm away each other by a microtome. The sections were deposited onto CaF_2_ optical windows (1 mm thick, 13 mm diameter) and let air-dry for 30 min.

### Quantification and statistical analysis

The statistical analysis was performed using GraphPad Prism version 8.00 (GraphPad Software) and SPSS statistical software version 25.0 (IBM Corporation). One-way analysis of variance (ANOVA) and Tukey’s multiple comparisons test evaluated the differences among groups examined by both PhC-microCT and FTIRI measurements. Correlation analysis between the collagen bundles morphometric indices, FTIRI features, and clinicopathological stage were explored by Spearman rank correlation analysis. A P-value of <0.05 was accepted as statistically significant for all tests. ∗P < 0.05; ∗∗P < 0.01; ∗∗∗P < 0.001; ∗∗∗∗P < 0.0001.
